# Access to gender-affirming hormones during adolescence and mental health outcomes among transgender adults

**DOI:** 10.1371/journal.pone.0261039

**Published:** 2022-01-12

**Authors:** Jack L. Turban, Dana King, Julia Kobe, Sari L. Reisner, Alex S. Keuroghlian

**Affiliations:** 1 Division of Child & Adolescent Psychiatry, Stanford University School of Medicine, Stanford, California, United States of America; 2 The Fenway Institute, Boston, Massachusetts, United States of America; 3 Division of Endocrinology, Diabetes, and Hypertension, Brigham and Women’s Hospital, Boston, Massachusetts, United States of America; 4 Department of Medicine, Harvard Medical School, Boston, Massachusetts, United States of America; 5 Department of Epidemiology, Harvard T.H. Chan School of Public Health, Boston, Massachusetts, United States of America; 6 Department of Psychiatry, Harvard Medical School, Boston, Massachusetts, United States of America; 7 Department of Psychiatry, Massachusetts General Hospital, Boston, Massachusetts, United States of America; Callen-Lorde Community Health Center, UNITED STATES

## Abstract

**Objective:**

To examine associations between recalled access to gender-affirming hormones (GAH) during adolescence and mental health outcomes among transgender adults in the U.S.

**Methods:**

We conducted a secondary analysis of the 2015 U.S. Transgender Survey, a cross-sectional non-probability sample of 27,715 transgender adults in the U.S. Using multivariable logistic regression adjusting for potential confounders, we examined associations between access to GAH during early adolescence (age 14–15), late adolescence (age 16–17), or adulthood (age ≥18) and adult mental health outcomes, with participants who desired but never accessed GAH as the reference group.

**Results:**

21,598 participants (77.9%) reported ever desiring GAH. Of these, 8,860 (41.0%) never accessed GAH, 119 (0.6%) accessed GAH in early adolescence, 362 (1.7%) accessed GAH in late adolescence, and 12,257 (56.8%) accessed GAH in adulthood. After adjusting for potential confounders, accessing GAH during early adolescence (aOR = 0.4, 95% CI = 0.2–0.6, p < .0001), late adolescence (aOR = 0.5, 95% CI = 0.4–0.7, p < .0001), or adulthood (aOR = 0.8, 95% CI = 0.7–0.8, p < .0001) was associated with lower odds of past-year suicidal ideation when compared to desiring but never accessing GAH. In post hoc analyses, access to GAH during adolescence (ages 14–17) was associated with lower odds of past-year suicidal ideation (aOR = 0.7, 95% CI = 0.6–0.9, p = .0007) when compared to accessing GAH during adulthood.

**Conclusion:**

Access to GAH during adolescence and adulthood is associated with favorable mental health outcomes compared to desiring but not accessing GAH.

## Introduction

A recent representative sample of adolescents in the United States (U.S.) found that 1.8% identified as transgender [1]. Unfortunately, these young people face a range of mental health disparities, including elevated rates of anxiety, depression, and suicide attempts [2]. Suicide attempt prevalence among transgender young adults has been estimated to be as high as 40% [3]. These disparities are generally thought to be due to two processes: gender minority stress and dysphoria related to one’s body developing in ways that are incongruent with one’s gender identity (i.e., a person’s psychological sense of their own gender) [2].

Gender minority stress refers to the ways in which society’s mistreatment of transgender people results in worse mental and physical health outcomes. This includes distal factors (gender-related discrimination, gender-related rejection, gender-related victimization, and non-affirmation of gender identity), as well as subsequent proximal factors (internalized transphobia, negative expectations, and concealment) [4]. Creating safe and affirming social environments for transgender adolescents is thus considered paramount in preventing adverse mental health outcomes [5].

In addition to creating safe and affirming environments, care for transgender people often involves the provision of gender-affirming medical interventions to alleviate the psychological distress related to one’s body developing in ways that do not align with one’s gender identity [6, 7]. This may include pubertal suppression for younger adolescents and gender-affirming hormones (GAH, e.g., estrogen and testosterone) from adolescence onward to induce physical changes that match the person’s gender identity [6–8]. Some adolescents may undergo gender-affirming surgery to reduce psychological distress [9, 10]. Of note, past Endocrine Society guidelines recommended that GAH not be considered until an adolescent reaches age 16 [11]. More recent guidelines state that initiation of GAH can be considered as early as age 14, to allow transgender adolescents to undergo puberty at ages more comparable to their peers, and to reduce the risk of delayed bone development due to prolonged pubertal suppression [7]. In this article, we therefore consider two age groups of adolescents who initiated GAH: those who started GAH during late adolescence (i.e., between their 16^th^ and 18^th^ birthdays) and those who started GAH during early adolescence (i.e., between their 14^th^ and 16^th^ birthdays).

To date, there have been six longitudinal cohort studies examining the impact of GAH initiation during adolescence on mental health [12–17]. These studies have generally found improvement in mental healh following adolescent GAH initiation, including decreases in internalizing psychopathology, improved general wellbeing, and decreased suicidality. Of note, these studies did not include a comparison group of adolescents who did not access GAH. Furthermore, these studies did not examine separately those who initiated GAH during early or late adolescence, nor did they compare initiation of GAH during adolescence with initiation of GAH during adulthood.

The impact of GAH initiated in adolescence on the mental health of transgender adults is of particular policy relevance today, as several U.S. states have introduced legislation to limit access to GAH for transgender adolescents, despite opposition from major medical organizations including The American Medical Association, The American Academy of Pediatrics, The American Psychiatric Association, The American Academy of Child & Adolescent Psychiatry, The Endocrine Society, The Pediatric Endocrine Society, and others [18]. This is an area of active policy debate where additional quantitative data are needed to guide policy decisions. Parents of transgender youth have been particularly concerned about these restrictive legislative efforts, with a parent in one recent qualitative sudy noting, “this could mean death for my child” [19].

The current study uses the largest survey of transgender people conducted to date to examine associations between recalled access to GAH during early adolescence (ages 14–15), late adolescence (ages 16–17), or adulthood (age ≥18), and adult mental health outcomes including measures of suicidality. It is the first study of GAH initiation during adolescence that includes a comparison group of those who desired but never accessed GAH. It is also the first to compare access to GAH during adolescence with access to GAH during adulthood. Given the large sample size, we were able to adjust for a wide range of potential confounding variables known to be associated with mental health outcomes for transgender people. We hypothesized that access to GAH during both early and late adolescence would be associated with more favorable mental health outcomes reported in adulthood, when compared to desiring but never accessing GAH.

## Methods

### Study population

The 2015 U.S. Transgender Survey (USTS) is the largest existing dataset of transgender people to date [3]. The cross-sectional non-probability survey was conducted between August and September of 2015. Transgender adults ages 18 years or older were recruited in collaboration with over 400 community organizations and completed measures online. The final survey had 27,715 participants from all 50 U.S. states, as well as Washington D.C., Puerto Rico, and U.S. territories abroad. Because not all transgender people necessarily desire GAH, we restricted the current study to participants who reported ever desiring GAH for gender affirmation, as this is a more clinically relevant group. This was assessed by choosing “hormone therapy/HRT (an acronym for ‘Hormone Replacement Therapy’)” in response to the question, “Have you ever wanted any of the health care listed below for your gender identity or gender transition? (Mark all that apply).” Options included “counseling/therapy,” “hormone treatment/HRT,” “puberty blocking hormones (usually used by youth ages 9–16),” and “none of the above.” This resulted in inclusion of 21,598 participants.

### Ethical considerations

The protocol for the USTS was approved by the University of California Los Angeles Institutional Review Board. The protocol for the current study was reviewed by The Fenway Institute Institutional Review Board. All participants provided informed consent for study participation.

### Age of initiation of GAH

Participants were divided into four categories. The first group, “wanted but never accessed GAH” (No GAH), reported never accessing GAH despite desiring these medications. The second group consisted of participants who reported they first accessed GAH during early adolescence, defined as the period between their 14^th^ and 16^th^ birthdays (GAH 14–15), which corresponds to the age group most recently added to the Endocrine Society Guidelines [7]. The third group consisted of participants who reported they first accessed GAH during late adolescence, defined as the period between their 16^th^ and 18^th^ birthdays (GAH 16–17), corresponding to the narrower age group in the prior, 2009 Endocrine Society Guidelines [11]. The fourth group consisted of participants who reported they first accessed GAH after their 18^th^ birthday (GAH ≥ 18).

### Outcomes

Severe psychological distress in the month prior to the survey was defined as a score ≥ 13 on the Kessler-6 Psychological Distress Scale [20]. Binge drinking in the month prior to the survey was defined as drinking 5 or more standard alcoholic drinks on a single occasion, a threshold for use in research with transgender adults that has been discussed in prior reports [21]. Lifetime illicit drug use (excluding marijuana) was also assessed as a binary “yes” or “no” self-report outcome. Measures of suicidality were examined, including suicidal ideation during the year prior to the survey, suicidal ideation with plan during the prior year, suicide attempt during the prior year, and suicide attempt requiring hospitalization during the prior year [8]. All suicidality measures were binary outcome variables in which participants reported “yes” or “no.”

### Demographic and other potential confounding variables

Demographic and other potential confounding variables that are known to be associated with adverse mental health outcomes among transgender people were collected for participants and included age at time of survey completion (U.S. census categories), gender identity, sex assigned at birth, sexual orientation, race/ethnicity (U.S. census categories), level of family support for gender identity (unsupportive, neutral, supportive, or not asked because participant had not disclosed being transgender to their family) [22], relationship status, level of education, employment status, household income, having ever received pubertal suppression (e.g., treatment with gonadotropin-releasing hormone agonists) [8], having ever been exposed to gender identity conversion efforts [23], and having experienced any harassment based on gender identity in K-12 (verbal, physical, or sexual) [5].

### Statistical analyses

All statistical analyses were performed with SAS 9.4. The data in the analytic sample had minimal missing data for both exposure and outcome variables. Each control variable had under 8% missing data within all comparison groups. Therefore no imputation was performed, since listwise deletion with missingness as high as 10% can be acceptable under particular assumptions of missingness [24].

Analyses were performed for the three age groups of participants who accessed GAH and participants who desired but never accessed GAH, on demographic variables listed above. Variables were analyzed with Rao-Scott χ^2^ tests. Logistic regression tests were used to identify demographics and other potential confounding variables associated with each outcome.

Multivariable logistic regression was then performed, comparing mental health outcomes for participants who reported access to GAH during early adolescence, late adolescence, or adulthood with those for participants who desired but never accessed GAH. Models were fit to test associations with mental health outcomes, after adjusting for demographic and potential confounding variables that were found to be associated with each outcome. All hypothesis tests were 2-sided. The percentage decrease in adjusted odds for the outcomes was calculated from the model coefficients for each age group.

In order to account for multiple comparisons, a modified Bonferroni correction was applied for the approximately 50 comparisons performed. A significance threshold of 0.001 (.05/50) was used for our analyses.

After all aforementioned analyses were completed, we identified further analyses of interest that were not included in the original study design, and therefore not included in the Bonferroni correction. In these post hoc analyses, we compared access to GAH during adolescence (ages 14–17) to access during adulthood (ages ≥18), and access to GAH during early adolescence (ages 14–15) to access during late adolescence (ages 16–17).

## Results

### Demographic differences & potential confounding variables

In total, 21,598 participants (77.9%) reported ever desiring GAH. Of these, 8,860 (41.0%) never accessed GAH, 119 (0.6%) reported access to GAH in early adolescence, 362 (1.7%) reported access to GAH in late adolescence, and 12,257 (56.8%) reported access to GAH in adulthood. Significant differences were found based on age at time of study participation, gender identity, sex assigned at birth, sexual orientation, race/ethnicity, family support of gender identity, relationship status, level of education, employment status, household income, having ever received pubertal suppression, having ever been exposed to gender identity conversion efforts, and having experienced verbal, physical, or sexual harassment based on gender identity in K-12 (Table 1).

**Table 1:** Sample demographics.

Total N = 21,598		No GAH	GAH 14–15	GAH 16–17	GAH ≥ 18	
		n = 8860	n = 119	n = 362	n = 12257	
		n (%)	n (%)	n (%)	n (%)	p
Age (Census)						**<0.001**
	18–24	5315 (60.0)	75 (63.03)	297 (82.04)	2856 (23.30)	
	25–44	2653 (29.9)	23 (19.33)	54 (14.92)	6285 (51.28)	
	45–64	753 (8.5)	19 (15.97)	11 (3.04)	2660 (21.70)	
	65+	139 (1.57)	2 (1.68)	0 (0.00)	456 (3.72)	
Gender Identity						**<0.001**
	Trans man / male	02620 (29.57)	00048 (40.34)	00214 (59.12)	04713 (38.45)	
	Trans woman / female	02324 (26.23)	00054 (45.38)	00109 (30.11)	06340 (51.73)	
	AFAB GQ/NB	02829 (31.93)	00013 (10.92)	00035 (9.67)	00834 (6.80)	
	AMAB GQ/NB	00766 (8.65)	00004 (3.36)	00004 (1.10)	00330 (2.69)	
	Other	00321 (3.62)	00000 (0.00)	00000 (0.00)	00040 (0.33)	
Sex Assigned at Birth						**<0.001**
	Female	05475 (61.79)	00061 (51.26)	00249 (68.78)	05561 (45.37)	
	Male	03385 (38.21)	00058 (48.74)	00113 (31.22)	06696 (54.63)	
Sexual Orientation						**<0.001**
	Asexual	01220 (13.77)	00006 (5.04)	00022 (6.08)	00771 (06.29)	
	Bisexual	01391 (15.70)	00007 (5.88)	00056 (15.47)	01900 (15.50)	
	Gay/Lesbian/Same Gender Loving	01337 (15.09)	00022 (18.49)	00064 (17.68)	02535 (20.68)	
	Heterosexual/Straight	00743 (8.39)	00031 (26.05)	00071 (19.61)	02019 (16.47)	
	Pansexual	01875 (21.16)	00021 (17.65)	00066 (18.23)	01877 (15.31)	
	Queer	01573 (17.75)	00019 (15.97)	00058 (16.02)	02525 (20.60)	
	Other	00721 (08.14)	00013 (10.92)	00025 (6.91)	00630 (5.14)	
Race / Ethnicity						**<0.001**
	Alaska Native/American Indian	00105 (1.19)	00002 (1.68)	00003 (0.83)	00149 (1.22)	
	Asian/Native Hawaiian/Pacific Islander	00273 (3.08)	00008 (6.72)	00010 (2.76)	00292 (2.38)	
	Biracial/Multiracial	00475 (5.36)	00009 (7.56)	00027 (7.46)	00571 (4.66)	
	Black/African American	00210 (2.37)	00011 (9.24)	00016 (4.42)	00378 (3.08)	
	Latin/Hispanic	00499 (5.63)	00008 (6.72)	00025 (6.91)	00572 (4.67)	
	White/Middle Eastern/North African	07298 (82.37)	00081 (68.07)	00281 (77.62)	10295 (83.99)	
Family Support of Gender Identity						**<0.001**
	Not Asked (Not Out to Family as Transgender)	03067 (34.64)	00003 (2.52)	00015 (4.14)	00901 (7.36)	
	Neutral	01564 (17.66)	00012 (10.08)	00032 (8.84)	01980 (16.16)	
	Supportive	02904 (32.80)	00091 (76.47)	00291 (80.39)	07321 (59.77)	
	Unsupportive	01319 (14.90)	00013 (10.92)	00024 (6.63)	02047 (16.71)	
	Missing	6 (0.07)	0 (0.00)	0 (00.00)	8 (0.08)	
Relationship Status						**<0.001**
	Partnered	04028 (46.90)	00049 (43.36)	00135 (38.03)	06257 (52.99)	
	Unpartnered	04560 (53.10)	00064 (56.64)	00220 (61.97)	05551 (47.01)	
	Other	272 (3.07)	6 (5.04)	7 (1.93)	449 (3.66)	
Education						**<0.001**
	Bachelor’s degree or higher	02219 (25.05)	00023 (19.33)	00048 (13.26)	05911 (48.23)	
	Some college (no degree)/Associate’s	04555 (51.41)	00061 (51.26)	00171 (47.24)	05199 (42.42)	
	High school grad (including GED)	01617 (18.25)	00023 (19.33)	00099 (27.35)	00975 (7.95)	
	Less than high school	00469 (5.29)	00012 (10.08)	00044 (12.15)	00172 (1.40)	
Employment Status						**<0.001**
	Employed	05213 (59.10)	00060 (50.85)	00189 (52.50)	08788 (72.01)	
	Out of the labor force	02038 (23.10)	00039 (33.05)	00108 (30.00)	02283 (18.71)	
	Unemployed	01570 (17.80)	00019 (16.10)	00063 (17.50)	01133 (9.28)	
	Excluded (status unclear)	4 (0.05)	0 (0)	2 (00.55)	2 (0.02)	
	Missing	35 (0.40)	1 (0.48)	0 (0)	51 (0.42)	
Household Income						**<0.001**
	$1 to $9,999	01163 (14.75)	00016 (14.81)	00041 (12.65)	01160 (10.10)	
	$10,000 to $24,999	01714 (21.73)	00013 (12.04)	00053 (16.36)	02252 (19.62)	
	$100,000 or more	01136 (14.40)	00023 (21.30)	00079 (24.38)	02064 (17.98)	
	$25,000 to $49,999	01717 (21.77)	00028 (25.93)	00059 (18.21)	02652 (23.10)	
	$50,000 to $100,000	01772 (22.47)	00024 (22.22)	00071 (21.91)	03035 (26.44)	
	No income	00385 (4.88)	00004 (3.70)	00021 (6.48)	00317 (2.76)	
	Excluded	275 (3.10)	7 (5.88)	11 (3.04)	313 (2.55)	
	Missing	698 (7.88)	4 (3.36)	27 (7.46)	464 (3.79)	
Ever Received Pubertal Suppression						**<0.001**
	Yes	00031 (0.36)	00041 (34.45)	00044 (12.15)	00221 (01.80)	
	No	08659 (99.64)	00078 (65.55)	00318 (87.85)	12036 (98.20)	
	Missing	00170 (1.92))	0 (0.00))	0 (0.00))	0 (0.00))	
Ever Experienced Gender Identity Conversion Efforts						**<0.001**
	Yes	00998 (11.28)	00031 (26.27)	00092 (25.48)	02208 (18.03)	
	No	07852 (88.72)	00087 (73.73)	00269 (74.52)	10037 (81.97)	
	Missing	10 (0.11)	1 (0.84)	1 (0.28)	12 (0.10)	
K-12 Harassment						**<0.001**
	Verbal, physical or sexual	2026 (22.9)	80 (67.2)	226 (62.4)	2612 (21.3)	
	None	6834 (77.1)	39 (32.8)	136 (37.6)	9645 (78.7)	

Descriptive statistics for transgender adults in the U.S. who ever desired gender-affirming hormones (GAH) for their gender identity or gender transition, comparing those who never accessed this treatment (No GAH), those who accessed GAH between their 14^th^ and 16^th^ birthdays (GAH 14–15), those who accessed GAH after their 16th birthday and before their 18th birthday (GAH 16–18) and those who accessed GAH after their18th birthday (GAH ≥ 18).

Abbreviations: AFAB (assigned female at birth), AMAB (assigned male at birth), GQ/NB (gender queer or non-binary).

### GAH during early adolescence

The median age of participants who reported accessing GAH during early adolescence was 21.0 (IQR 18.0–35.0). After adjusting for demographic and potential confounding variables, recalled access to GAH during early adolescence was associated with lower odds of past-month severe psychological distress (aOR = 0.3, 95% CI = 0.2–0.4, p < .0001) and past-year suicidal ideation (aOR = 0.4, 95% CI = 0.2–0.6, p < .001) when compared to desiring GAH but never accessed them. For participants who recalled GAH access in early adolescence, these results represent a 222% decrease in adjusted odds for past-month severe psychological distress and a 135% decrease for past-year suicidal ideation. We detected no difference for other mental health variables measured (Table 2).

**Table 2:** Outcomes for participants who accessed gender-affirming hormones (estrogen or testosterone).

	Participants who Accessed GAH											
	N = 12,598											
	Accessed GAH at Age 14 or 15				Accessed GAH at Age 16 or 17				Accessed GAH at Age ≥ 18			
	n = 119				n = 362				n = 12257			
	OR (95% CI)	p	aOR (95% CI)	p	OR (95% CI)	p	aOR (95% CI)	p	OR (95% CI)	p	aOR (95% CI)	p
**Suicidality (Past 12 months)**												
Past-year suicidal ideation ^a^	0.5 (0.3–0.7)	.0001	0.4 (0.2–0.6)	**< .0001**	1.0 (0.8–1.2)	.73	0.5 (0.4–0.7)	**< .0001**	0.5 (0.5–0.6)	< .0001	0.8 (0.7–0.8)	**< .0001**
Past-year suicidal ideation with plan ^b^	1.3 (0.8–2.4)	.31	0.8 (0.4–1.6)	.58	1.1 (0.9–1.5)	.41	0.9 (0.7–1.2)	.49	0.8 (0.8–0.9)	< .0001	0.9 (0.8–1.0)	.09
Past-year suicide attempt ^c^	1.0 (0.5–2.2)	.99	0.4 (0.2–1.1)	.08	1.4 (1.0–2.0)	.04	0.9 (0.6–1.4)	.79	0.8 (0.8–0.9)	.002	1.0 (0.9–1.1)	.89
Past-year suicide attempt requiring inpatient hospitalization ^d^	––	––	––	––	2.2 (1.2–4.0)	.01	2.2 (1.2–4.2)	.01	1.4 (1.1–1.7)	.002	1.2 (0.9–1.5)	.26
**Mental Health & Substance Use**												
Past-month severe psychological distress (K6 ≥ 13) ^c^	0.5 (0.3–0.7)	.0004	0.3 (0.2–0.4)	**< .0001**	0.6 (0.5–0.8)	< .0001	0.3 (0.3–0.4)	**< .0001**	0.4 (0.3–0.4)	< .0001	0.6 (0.5–0.6)	**< .0001**
Past-month binge drinking ^e^	1.6 (1.1–2.3)	.02	1.6 (1.0–2.4)	.04	0.8 (0.6–1.1)	.17	0.9 (0.6–1.1)	.27	1.2 (1.1–1.2)	< .0001	1.2 (1.1–1.3)	**< .0001**
Lifetime illicit drug use ^f^	1.8 (1.2–2.6)	.003	1.5 (1.0–2.2)	.08	1.2 (1.0–1.6)	.08	1.3 (1.0–1.6)	.07	2.1 (1.9–2.2)	< .0001	1.7 (1.6–1.8)	**< .0001**

Mental health outcomes of transgender adults who recalled access to gender-affirming hormones (GAH) during various age groups. Reference group for all analyses is participants who desired GAH but did not access them. All models adjusted for age, partnership status, employment status, K-12 harassment, and having experienced gender identity conversion efforts.

Abbreviations: OR (odds ratio), aOR (adjusted odds ratio), 95% CI (95% confidence interval).

^a^ Model also adjusted for gender identity, sex assigned at birth, sexual orientation, race/ethnicity, family support of gender identity, educational attainment, and total household income.

^b^ Model also adjusted for sexual orientation, race/ethnicity, educational attainment, and total household income.

^c^ Model also adjusted for gender identity, sex assigned at birth, sexual orientation, race/ethnicity, family support of gender identity, educational attainment, total household income, and having received pubertal suppression.

^d^ Model also adjusted for family support of gender identity. Only one participant in the GAH < 16 group endorsed a past–year suicide attempt requiring inpatient hospitalization, precluding calculation of an aOR for this outcome.

^e^ Model also adjusted for gender identity, sex assigned at birth, sexual orientation, family support of gender identity, educational attainment, and total household income.

^f^ Model also adjusted for gender identity, sex assigned at birth, sexual orientation, race/ethnicity, family support of gender identity, and educational attainment.

### GAH during late adolescence

The median age of participants who reported accessing GAH during late adolescence was 19.0 (IQR 18.0–22.0). After adjusting for demographic and potential confounding variables, recalled access to GAH during late adolescence was associated with lower odds of past-month severe psychological distress (aOR = 0.3, 95% CI = 0.3–0.4, p < .0001) and past-year suicidal ideation (aOR = 0.5, 95% CI = 0.4–0.7, p < .0001) when compared to desiring GAH but never accessing them. These results represent a 153% decrease in the adjusted odds for past-month severe psychological distress and a 62% decrease for past-year suicide ideation. We detected no difference for other mental health variables measured (Table 2).

### GAH during adulthood

The median age of participants who reported accessing GAH during adulthood was 31.0 (IQR 25.0–45.0). After adjusting for demographic and potential confounding variables, participants who recalled access to GAH during adulthood had lower odds of past-month severe psychological distress (aOR = 0.6, 95% CI = 0.5–0.6, p < .0001) and past-year suicidal ideation (aOR = 0.8, 95% CI = 0.7–0.8, p < .0001) when compared to those who desired GAH but never accessed them. Access to GAH during adulthood was associated with an 81% decrease in adjusted odds of past-month severe psychological distress and a 21% decrease in past-year suicidal ideation. Access to GAH during adulthood was also associated with greater odds of past-month binge drinking (aOR = 1.2, 95% CI = 1.1–1.3, p < .0001) and lifetime illicit drug use (aOR = 1.7, 95% CI = 1.6–1.8, p < .0001) when compared to desiring but never accessing GAH. Results indicated an adjusted odds increase of 20% for past-month binge drinking and 70% increase for lifetime illicit drug use. We detected no difference for other mental health variables measured (Table 2).

### Raw frequencies of outcome variables

Raw frequencies for outcome variables are shown in Table 3.

**Table 3:** Raw outcome frequencies of mental health outcomes.

Total N = 21,598	No GAH	GAH 14–15	GAH 16–17	GAH ≥ 18
	n = 8860	n = 119	n = 362	n = 12257
	n (%)	n (%)	n (%)	n (%)
**Suicidality (Past 12 months)**				
Past-year suicidal ideation	5144 (58.1)	48 (40.3)	40 (33.6)	5237 (42.7)
Past-year suicidal ideation with plan	2731 (30.8)	29 (24.3)	39 (32.8)	02537 (20.7)
Past-year suicide attempt	853 (9.6)	8 (6.7)	40 (33.6)	756 (6.2)
Past-year suicide attempt requiring inpatient hospitalization	220 (2.5)	1 (0.8)	40 (33.6)	247 (2.0)
**Mental Health & Substance Use**				
Past-month severe psychological distress (K6 ≥ 13)	4545 (51.3)	40 (33.6)	145 (40.1)	3419 (27.9)
Past-month binge drinking	2083 (23.5)	39 (32.8)	74 (20.4)	3214 (26.2)
Lifetime illicit drug use	1918 (21.6)	40 (33.6)	93 (25.7)	4455 (36.3)

### Post hoc analyses

#### GAH during adolescence vs. GAH during adulthood

After adjusting for demographic and potential confounding variables, access to GAH during adolescence (ages 14–17) was associated with lower odds of past-month severe psychological distress (aOR = 0.6, 95% CI = 0.5–0.8, p < .0001), past-year suicidal ideation (aOR = 0.7, 95% CI = 0.6–0.9, p = .0007), past-month binge drinking (aOR = 0.7, 95% CI = 0.5–0.9, p = .001), and lifetime illicit drug use (aOR = 0.7, 95% CI = 0.5–0.8, p = .0003) when compared to access to GAH during adulthood. We detected no difference for other mental health variables measured (Table 4).

**Table 4:** Outcomes for participants who accessed gender-affirming hormones (estrogen or testosterone).

	Accessed GAH at Age 14–17				Accessed GAH at Age 14 or 15			
	(compared to GAH access at age ≥ 18)				(compared to GAH access at age 16 or 17)			
	n = 481				n = 119			
	OR (95% CI)	p	aOR (95% CI)	p	OR (95% CI)	p	aOR (95% CI)	p
**Suicidality (Past 12 months)**								
Past-year suicidal ideation ^a^	1.5 (1.3–1.8)	< .0001	0.7 (0.6–0.9)	**.0007**	0.5 (0.3–0.8)	.002	0.7 (0.4–1.2)	.16
Past-year suicidal ideation with plan ^b^	1.4 (1.1–1.8)	.009	1.1 (0.8–1.5)	.51	1.2 (0.6–2.3)	.58	1.0 (0.5–1.9)	.88
Past-year suicide attempt^c^	1.6 (1.2–2.2)	.003	1.0 (0.7–1.4)	.82	0.7 (0.3–1.6)	.40	0.4 (0.1–1.3)	.12
Past-year suicide attempt requiring inpatient hospitalization ^d^	1.3 (0.7–2.3)	.35	1.7 (0.9–3.2)	.08	0.2 (0.0–1.6)	.13	0.2 (0.0–2.1)	.19
**Mental Health & Substance Use**								
Past-month severe psychological distress (K6 ≥ 13) ^c^	1.7 (1.4–2.0)	< .0001	0.6 (0.5–0.8)	**< .0001**	0.8 (0.5–1.2)	.26	0.7 (0.4–1.3)	.30
Past–month binge drinking ^e^	0.9 (0.7–1.1)	.17	0.7 (0.5–0.9)	**.001**	1.9 (1.2–3.0)	.006	2.0 (1.2–3.5)	.01
Lifetime illicit drug use ^f^	0.7 (0.5–0.8)	< .001	0.7 (0.5–0.8)	**.0003**	1.4 (0.9–2.3)	.10	1.0 (0.6–1.7)	.98

All models adjusted for age, partnership status, employment status, K-12 harassment, and having experienced gender identity conversion efforts.

Abbreviations: OR (odds ratio), aOR (adjusted odds ratio), 95% CI (95% confidence interval).

^a^ Model also adjusted for gender identity, sex assigned at birth, sexual orientation, race/ethnicity, family support of gender identity, educational attainment, and total household income.

^b^ Model also adjusted for sexual orientation, race/ethnicity, educational attainment, and total household income.

^c^ Model also adjusted for gender identity, sex assigned at birth, sexual orientation, race/ethnicity, family support of gender identity, educational attainment, total household income, and having received pubertal suppression.

^d^ Model also adjusted for family support of gender identity.

^e^ Model also adjusted for gender identity, sex assigned at birth, sexual orientation, family support of gender identity, educational attainment, and total household income.

^f^ Model also adjusted for gender identity, sex assigned at birth, sexual orientation, race/ethnicity, family support of gender identity, and educational attainment.

#### Access to GAH during early vs. late adolescence

After adjusting for demographic and potential confounding variables, we detected no difference in odds of any mental health variables measured when comparing access to GAH during early adolescence with access to GAH during late adolescence (Table 4).

#### Lifetime but no past year suicidality

Due to the cross-sectional nature of the study, it was possible that we detected an association between favorable mental health outcomes and access to GAH because people with better mental health were more likely to be able to access GAH. Given that baseline mental health status could confound associations between access to GAH and mental health outcomes, in post hoc analyses we examined two outcome measures relevant to this question of temporality: lifetime but no past-year suicidal ideation, and lifetime but no past-year suicide attempt. We found that access to GAH in adulthood was associated with greater odds of lifetime but no past-year suicidal ideation (aOR = 1.4, 95% CI = 1.3–1.5, p < .0001) when compared to desiring but not accessing GAH (Table 5). The association of access to GAH during late adolescence with lifetime but no past year suicidal ideation (aOR = 1.4, 95% CI = 1.1–1.8, p = .005) was no longer significant after Bonferroni correction, though some have noted that Bonferroni adjustment may be overly conservative, suggesting that this finding may be considered significant [25].

**Table 5:** Lifetime but no past-year suicide ideation and attempts for participants who accessed gender-affirming hormones (estrogen or testosterone).

	Participants who Accessed GAH					
	N = 12,598					
	Accessed GAH at Age 14 or 15		Accessed GAH at Age 16 or 17		Accessed GAH at Age ≥ 18	
	n = 119		n = 362		n = 12,257	
	aOR (95% CI)	p	aOR (95% CI)	p	aOR (95% CI)	p
Lifetime suicidal ideation and no past-year ideation ^a^	1.3 (0.8–2.0)	.28	1.4 (1.1–1.8)	.005	1.4 (1.3–1.5)	**< .0001**
Lifetime suicide attempt and no past-year attempt ^b^	0.8 (0.5–1.2)	.24	0.7 (0.6–1.0)	.03	1.0 (0.9–1.1)	.67

Mental health outcomes of transgender adults who recalled access to gender-affirming hormones (GAH) during various age groups. Reference group for all analyses is participants who desired GAH but did not access them. Both models adjusted for age, partnership status, employment status, K-12 harassment, and having experienced gender identity conversion efforts.

^a^ Model also adjusted for gender identity, sex assigned at birth, sexual orientation, race/ethnicity, family support of gender identity, educational attainment, and total household income.

^b^ Model also adjusted for gender identity, sex assigned at birth, sexual orientation, race/ethnicity, family support of gender identity, educational attainment, total household income, and having received pubertal suppression.

## Discussion

In this large national cross-sectional non-probability study, transgender people who accessed GAH during early adolescence, late adolescence, or adulthood had better mental health outcomes when compared to those who desired but were unable to access GAH. Given the substantial mental health disparities faced by transgender people, these results are of particular importance [26].

For each time period of GAH initiation examined (early adolescence, late adolescence, and adulthood), access to GAH was associated with lower odds of past-year suicidal ideation and past-month severe psychological distress. When we compared participants who accessed GAH during adolescence (ages 14–17) with those who accessed GAH during adulthood (18+), participants who accessed GAH earlier had better mental health outcomes, including lower odds of past-year suicidal ideation, past-month severe psychological distress, past-month binge drinking, and lifetime illicit drug use. These results argue against waiting until adulthood to offer GAH to transgender adolescents and suggest that doing so may put patients at greater mental health risk.

The current study has a few advantages over past published studies in this area. While past studies have not included a comparison group of people who did not access GAH and were also underpowered to adjust for potential cofounders, this large sample size enabled comparison of participants who reported access to GAH to those who desired but did not access GAH, while adjusting for a wide range of potential confounding variables known to be associated with mental health outcomes for transgender people.

One unexpected finding was that participants who initiated GAH during adulthood, compared to those who desired but never accessed GAH, had greater odds of past-month binge drinking and lifetime illicit substance use. Transgender people often become more socially engaged following the increased confidence that results from gender affirmation, which may partly explain these results [27]. Given the high prevalence of substance use disorders in this population, clinicians ought to routinely screen for substance use disorders among transgender people, and researchers ought to focus on development of culturally responsive substance use disorder prevention and treatment interventions with transgender communities [27].

Notably, even participants who recalled access to GAH had high rates of past-year suicidal ideation. Though access to GAH during adolescence appears to be related to more favorable mental health outcomes, transgender people face a range of other psychosocial stressors that contribute to chronic minority stress, including but not limited to employment discrimination, lack of safe access to public facilities, and physical violence [4]. Future epidemiological and interventional research is needed to understand and address chronic minority stress among transgender people who access GAH as well as those who do not. For transgender adolescents, creating safe and affirming school environments appears to be of particular importance [28], in addition to providing gender-affirming medical care, as well as psychological, legal and surgical gender affirmation as needed [6].

This study also suggests that a large proportion of transgender people desire but never access GAH. Though prevalence in a non-probability sample should be interpreted with caution, 41% of those who desired GAH in this study reported that they were unable to access them. Barriers to accessing prescribed GAH, in addition to leaving many without treatment, may also drive use of non-prescribed GAH, which is highly prevalent and associated with stigmatizing healthcare policies [29]. Future studies ought to examine if non-prescribed GAH use, when compared to prescribed GAH, is linked to worse mental health outcomes or adverse physical health outcomes (e.g., blood clot risk from estradiol use without standard medical monitoring).

### Strengths and limitations

Strengths of this study include its large sample size and broad geographic representation within the U.S. The large sample size enabled adjustment for a wide range of potential confounding variables. Limitations include its non-probability cross-sectional design, which reduces generalizability and limits determination of causality. It is possible that people with better mental health status at baseline are more likely to be able to access GAH, thus confounding associations between GAH access and adult mental health outcomes measured: we therefore examined lifetime but no past-year suicidal ideation as an outcome, with results suggesting a lack of reverse causation due to such confounding. Nonetheless, this method is imperfect for investigating mental health changes following GAH, and future longitudinal studies are needed. Longitudinal waitlist control studies would be of particular value. Though a randomized controlled trial would help determine causality, many have noted that such a trial design is unethical in this context [2]. Age of GAH initiation reported by participants at time of data collection is vulnerable to recall bias. It is possible that participants in older age cohorts (45–65; 65+) were more vulnerable to recall bias; in our clinical experience, however, starting GAH is a major event in one’s life, making it less susceptible to recall bias than more routine events [30]. It was unexpected that the median age at time of survey completion for participants who recalled accessing GAH in early adolescence was older than for those in the late adolescence group, which may be indicative of recall bias. Of note, though it is often presumed that GAH were not offered to adolescents in the U.S. until the past three decades, recent historical analyses have pointed out that adolescents have been receiving GAH as early as the 1970s [31]. The 2015 USTS sample is younger, with fewer racial minorities, fewer heterosexual participants, and higher educational attainment when compared with probability samples of TGD people in the U.S [32]. Because all participants identified as non-cisgender, those who initiated GAH and subsequently identified as cisgender would not necessarily be represented in this study; existing literature, however, suggests that this is a rare occurrence [2, 33].

## Conclusion

This study found that transgender people who accessed GAH during early or late adolescence had a lower odds of past-month suicidal ideation and past-month severe psychological distress in adulthood, when compared to those who desired but did not access GAH, after adjusting for a range of potential confounding variables. The findings support updated 2017 recomendations from The Endocrine Society [7] and WPATH [6] that these medical interventions be made available for transgender adolescents. The results also provide additional evidence to suggest that legislation restricting transgender adolescents’ access to gender-affirming medical care would result in adverse mental health outcomes [18].
