# Correction: Access to gender-affirming hormones during adolescence and mental health outcomes among transgender adults

**DOI:** 10.1371/journal.pone.0287283

**Published:** 2023-06-12

**Authors:** Jack L. Turban, Dana King, Julia Kobe, Sari L. Reisner, Alex S. Keuroghlian

Concerns were raised, post-publication, regarding common publications of the handling Academic Editor and some of the authors. A second and independent member of the *PLOS ONE* Editorial Board has reevaluated the manuscript and reviews, and has confirmed that the article is scientifically sound and meets *PLOS ONE*’s Publication Criteria. They also confirmed that there are no concerns with the original reviews.

Additionally, after publication of this article [1], the authors discovered an error in the original manuscript. Specifically, throughout the article, the “early adolescence” group was mislabeled and inadvertently included all participants who accessed GAH prior to age 16, including some respondents who accessed GAH at ages younger than what is recommended in the most recent Endocrine Society Guidelines. Analyses for this “early adolescence” group have been updated to include only those who accessed GAH during the younger adolescent age group outlined by the most recent Endocrine Society guidelines (i.e., ages 13–15) [2]. The following specific errors have been corrected:

- The early adolescence group age (14–16) appears incorrectly throughout the article. The correct group age is (13–15). The Endocrine Society Guidelines note an age of 13.5, and the authors chose age 13 as a lower cutoff to include individuals who would have accessed GAH at this age.
- The number and percentage of the early adolescent group reporting access to GAH appears incorrectly through the article. The correct values are 99 (0.5%).
- The sample of individuals ever desiring GAH appears incorrectly throughout the article as 21,598. The correct value is 21,578, now that those reporting access to GAH younger than age 13 have been excluded.

The following sentence has been added to the first paragraph of the Methods section: We additionally excluded any participants who reported accessing GAH prior to age 13, as this would represent an age lower than the current threshold mentioned in the most recent Endocrine Society Guidelines.

Please see S1 Table for detailed in-text corrections and locations.

This error impacted Tables 1–5. In Table 3, there was an additional error in the third column, which has also been corrected here. Please see the correct tables below.

**Table 1:** Sample demographics.

Total N = 21,578		No GAH n = 8860	GAH 13–15 n = 99	GAH 16–17 n = 362	GAH ≥ 18 n = 12257	
		n (%)	n (%)	n (%)	n (%)	p
Age (Census)						**< .0001**
	18–24	5315 (60.00)	75 (63.03)	297 (82.04)	2856 (23.30)	
	25–44	2653 (29.94)	23 (19.33)	54 (14.92)	6285 (51.28)	
	45–64	753 (8.50)	13 (13.13)	11 (3.04)	2660 (21.70)	
	65+	139 (1.57)	1 (1.01)	0 (0.00)	456 (3.72)	
Gender Identity						**< .0001**
	Trans man / male	2620 (29.57)	46 (6.46)	214 (59.12)	4713 (38.45)	
	Trans woman / female	2324 (26.23)	38 (38.38)	109 (30.11)	6340 (51.73)	
	AFAB GQ/NB	2829 (31.93)	13 (13.13)	35 (9.67)	834 (6.80)	
	AMAB GQ/NB	766 (8.65)	2 (2.02)	4 (1.10)	330 (2.69)	
	Other	321 (3.62)	0 (0.00)	0 (0.00)	40 (0.33)	
Sex Assigned at Birth						**< .0001**
	Female	5475 (61.79)	59 (59.60)	00249 (68.78)	05561 (45.37)	
	Male	3385 (38.21)	40 (40.40)	00113 (31.22)	06696 (54.63)	
Sexual Orientation						**< .0001**
	Asexual	1220 (13.77)	4 (4.04)	22 (6.08)	771 (06.29)	
	Bisexual	1391 (15.70)	6 (6.06)	56 (15.47)	1900 (15.50)	
	Gay/Lesbian/Same Gender Loving	1337 (15.09)	13 (13.13)	64 (17.68)	2535 (20.68)	
	Heterosexual/Straight	743 (8.39)	27 (27.27)	71 (19.61)	2019 (16.47)	
	Pansexual	1875 (21.16)	20 (20.20)	66 (18.23)	1877 (15.31)	
	Queer	1573 (17.75)	18 (18.18)	58 (16.02)	2525 (20.60)	
	Other	721 (8.14)	11 (11.11)	25 (6.91)	630 (5.14)	
Race / Ethnicity						**< .0001**
	Alaska Native/American Indian	105 (1.19)	1 (1.01)	3 (0.83)	149 (1.22)	
	Asian/Native Hawaiian/Pacific Islander	273 (3.08)	6 (6.06)	10 (2.76)	292 (2.38)	
	Biracial/Multiracial	475 (5.36)	5 (5.05)	27 (7.46)	571 (4.66)	
	Black/African American	210 (2.37)	10 (10.10)	16 (4.42)	378 (3.08)	
	Latin/Hispanic	499 (5.63)	5 (5.05)	25 (6.91)	572 (4.67)	
	White/Middle Eastern/North African	7298 (82.37)	72 (72.73)	281 (77.62)	10295 (83.99)	
Family Support of Gender Identity						**< .0001**
	Not Asked (Not Out to Family as Transgender)	3067 (34.64)	2 (2.02)	00015 (4.14)	00901 (7.36)	
	Neutral	1564 (17.66)	8 (8.08)	32 (8.84)	1980 (16.16)	
	Supportive	2904 (32.80)	81 (81.82)	291 (80.39)	7321 (59.77)	
	Unsupportive	1319 (14.90)	8 (8.08)	24 (6.63)	2047 (16.71)	
	Missing	6 (0.07)	0 (0.00)	0 (00.00)	8 (0.08)	
Relationship Status						**< .0001**
	Partnered	4028 (46.90)	42 (42.42)	135 (38.03)	6257 (52.99)	
	Unpartnered	4560 (53.10)	54 (54.54)	220 (61.97)	5551 (47.01)	
	Other	272 (3.07)	4 (4.04)	7 (1.93)	449 (3.66)	
Education						**< .0001**
	Bachelor’s degree or higher	2219 (25.05)	14 (14.14)	48 (13.26)	5911 (48.23)	
	Some college (no degree)/Associate’s	4555 (51.41)	51 (51.52)	171 (47.24)	5199 (42.42)	
	High school grad (including GED)	1617 (18.25)	23 (23.23)	99 (27.35)	975 (7.95)	
	Less than high school	469 (5.29)	11 (11.11)	44 (12.15)	172 (1.40)	
Employment Status						**< .0001**
	Employed	5213 (59.10)	46(46.46)	189 (52.50)	8788 (72.01)	
	Out of the labor force	2038 (23.10)	35 (35.35)	108 (30.00)	2283 (18.71)	
	Unemployed	1570 (17.80)	17 (17.17)	63 (17.50)	1133 (9.28)	
	Excluded (status unclear)	4 (0.05)	0 (0.00)	2 (0.55)	2 (0.02)	
	Missing	35 (0.40)	1 (1.01)	0 (0.00)	51 (0.42)	
Household Income						**< .0001**
	$1 to $9,999	1163 (14.75)	14 (14.14)	41 (12.65)	1160 (10.10)	
	$10,000 to $24,999	1714 (21.73)	9 (9.09)	53 (16.36)	2252 (19.62)	
	$100,000 or more	1136 (14.40)	19 (19.19)	79 (24.38)	2064 (17.98)	
	$25,000 to $49,999	1717 (21.77)	23 (23.23)	59 (18.21)	2652 (23.10)	
	$50,000 to $100,000	1772 (22.47)	21 (21.21)	71 (21.91)	3035 (26.44)	
	No income	385 (4.88)	3 (3.03)	21 (6.48)	317 (2.76)	
	Excluded	275 (3.10)	6 (6.06)	11 (3.04)	313 (2.55)	
	Missing	698 (7.88)	4 (4.04)	27 (7.46)	464 (3.79)	
Ever Received Pubertal Suppression						**< .0001**
	Yes	31 (0.36)	31 (31.31)	44 (12.15)	221 (1.80)	
	No	8659 (99.64)	68 (68.69)	318 (87.85)	12036 (98.20)	
	Missing	170 (1.92))	0 (0.00))	0 (0.00))	0 (0.00))	
Ever Experienced Gender Identity Conversion Efforts						**< .0001**
	Yes	998 (11.28)	24 (24.24)	92 (25.48)	2208 (18.03)	
	No	7852 (88.72)	74 (74.75)	269 (74.52)	10037 (81.97)	
	Missing	10 (0.11)	1 (1.01)	1 (0.28)	12 (0.10)	
K-12 Harassment						**< .0001**
	Verbal, physical or sexual	2026 (22.90)	68 (68.69)	226 (62.43)	2612 (21.31)	
	None	6834 (77.13)	31 (31.31)	136 (37.60)	9645 (78.69)	

Descriptive statistics for transgender adults in the U.S. who ever wanted gender-affirming hormones (GAH) for their gender identity or gender transition, comparing those who never received this treatment (No GAH), those who accessed GAH between their 13^th^ and 16^th^ birthdays (GAH 13–15), those who accessed GAH after their 16th birthday and before their 18th birthday (GAH 16–18) and those who accessed GAH after 18th birthday (GAH ≥ 18).

Abbreviations: AFAB (assigned female at birth), AMAB (assigned male at birth), GQ/NB (gender queer or non-binary).

**Table 2:** Outcomes for those who received gender-affirming hormones (estrogen or testosterone).

** **	**Participants who Accessed GAH**											
	**N = 12,598**											
	**Accessed GAH at Ages 13–15**				**Accessed GAH at Ages 16 or 17**				**Accessed GAH at Age ≥ 18**			
	**n = 99**				**n = 362**				**n = 12257**			
	**OR**	**p**	**aOR**	**p**	**OR**	**p**	**aOR**	**p**	**OR**	**p**	**aOR**	**p**
	**(95% CI)**		**(95% CI)**		**(95% CI)**		**(95% CI)**		**(95% CI)**		**(95% CI)**	
**Suicidality (Past 12 months)**												
Past-year suicidal ideation ^a^	0.6	0.004	0.4	< .0001	1	0.73	0.5	< .0001	0.5	< .0001	0.8	< .0001
	(0.4–0.8)		(0.3–0.6)		(0.8–1.2)		(0.4–0.7)		(0.5–0.6)		(0.7–0.8)	
Past-year suicidal ideation with plan ^b^	1.2	0.51	0.8	0.55	1.1	0.41	0.9	0.49	0.8	< .0001	0.9	0.09
	(0.7–2.3)		(0.4–1.6)		(0.9–1.5)		(0.7–1.2)		(0.8–0.9)		(0.8–1.0)	
Past-year suicide attempt ^c^	0.8	0.64	0.4	0.06	1.4	0.04	0.9	0.79	0.8	0.002	1	0.89
	(0.3–1.9)		(0.1–1.1)		(1.0–2.0)		(0.6–1.4)		(0.8–0.9)		(0.9–1.1)	
Past-year suicide attempt requiring inpatient hospitalization ^d^	––	––	––	––	2.2	0.01	2.2	0.01	1.4	0.002	1.2	0.26
					(1.2–4.0)		(1.2–4.2)		(1.1–1.7)		(0.9–1.5)	
**Mental Health & Substance Use**												
Past-month severe psychological distress (K6 ≥ 13) ^c^	0.5	0.003	0.3	< .0001	0.6	< .0001	0.3	< .0001	0.4	< .0001	0.6	< .0001
	(0.4–0.8)		(0.2–0.5)		(0.5–0.8)		(0.3–0.4)		(0.3–0.4)		(0.5–0.6)	
Past-month binge drinking ^e^	1.6	0.04	1.6	0.04	0.8	0.17	0.9	0.27	1.2	< .0001	1.2	< .0001
	(1.0–2.4)		(1.0–2.6)		(0.6–1.1)		(0.6–1.1)		(1.1–1.2)		(1.1–1.3)	
Lifetime illicit drug use ^f^	1.7	0.01	1.6	0.05	1.2	0.08	1.3	0.07	2.1	< .0001	1.7	< .0001
	(1.1–2.6)		(1.0–2.5)		(1.0–1.6)		(1.0–1.6)		(1.9–2.2)		(1.6–1.8)	

Mental health outcomes of transgender adults who recalled access to gender-affirming hormones (GAH) during various age groups. Reference group for all analyses is those who desired GAH but did not receive them. All models adjusted for age, partnership status, employment status, K-12 harassment, and having experienced gender identity conversion efforts.

Abbreviations: OR (odds ratio), aOR (adjusted odds ratio), 95% CI (95% confidence interval).

a Model also adjusted for gender identity, sex assigned at birth, sexual orientation, race/ethnicity, family support of gender identity, educational attainment, and total household income.

b Model also adjusted for sexual orientation, race/ethnicity, educational attainment, and total household income.

c Model also adjusted for gender identity, sex assigned at birth, sexual orientation, race/ethnicity, family support of gender identity, educational attainment, total household income, having received pubertal suppression.

d Model also adjusted for family support of gender identity. Only one participant in the GAH < 16 group endorsed a past–year suicide attempt requiring inpatient hospitalization, precluding calculation of an aOR for this outcome.

e Model also adjusted for gender identity, sex assigned at birth, sexual orientation, family support of gender identity, educational attainment, and total household income.

f Model also adjusted for gender identity, sex assigned at birth, sexual orientation, race/ethnicity, family support of gender identity, and educational attainment.

**Table 3:** Raw outcome frequencies of mental health outcomes.

Total N = 21,578	No GAH n = 8860	GAH 13–15 n = 99	GAH 16–17 n = 362	GAH ≥ 18 n = 12257
	n (%)	n (%)	n (%)	n (%)
**Suicidality (Past 12 months)**				
Past-year suicidal ideation	5144 (58.1)	43 (43.4)	207 (57.2)	5237 (42.7)
Past-year suicidal ideation with plan	2731 (30.8)	25 (25.3)	116 (32.0)	02537 (20.7)
Past-year suicide attempt	853 (9.6)	6 (6.1)	46 (12.7)	756 (6.2)
Past-year suicide attempt requiring inpatient hospitalization	220 (2.5)	1 (1.0)	20 (5.5)	247 (2.0)
**Mental Health & Substance Use**				
Past-month severe psychological distress (K6 ≥ 13)	4545 (51.3)	35 (35.4)	145 (40.1)	3419 (27.9)
Past-month binge drinking	2083 (23.5)	32 (32.3)	74 (20.4)	3214 (26.2)
Lifetime illicit drug use	1918 (21.6)	32 (32.3)	93 (25.7)	4455 (36.3)

**Table 4:** Outcomes for those who received gender-affirming hormones (estrogen or testosterone).

	Accessed GAH at Ages 13–17 (compared to GAH at Age ≥ 18) n = 461				Accessed GAH at Ages 13–15 (compared to GAH at Ages 16 or 17) n = 99			
	OR (95% CI)	p	aOR (95% CI)	p	OR (95% CI)	p	aOR (95% CI)	p
**Suicidality (Past 12 months)**								
Past-year suicidal ideation ^a^	1.6 (1.3–1.9)	< .0001	0.7 (0.6–0.9)	.004	0.6 (0.4–0.9)	.02	0.8 (0.4–1.3)	.31
Past-year suicidal ideation with plan ^b^	1.4 (1.1–1.8)	.01	1.1 (0.8–1.5)	.52	1.1 (0.6–2.1)	.80	0.9 (0.4–1.9)	.79
Past-year suicide attempt ^c^	1.6 (1.1–2.1)	.006	1.0 (0.7–1.4)	.82	0.6 (0.2–1.4)	23	0.3 (0.1–1.1)	.07
Past-year suicide attempt requiring inpatient hospitalization ^d^	1.4 (0.8–2.5)	.26	1.8 (1.0–3.4)	.06	0.3 (0.0–2.4)	.24	0.3 (0.0–2.6)	.25
**Mental Health & Substance Use**								
Past-month severe psychological distress (K6 ≥ 13) ^c^	1.7 (1.4–2.1)	< .0001	0.6 (0.5–0.8)	**.0002**	0.8 (0.5–1.3)	.45	0.8 (0.5–1.5)	.50
Past–month binge drinking ^e^	0.8 (0.7–1.0)	.11	0.6 (0.5–0.8)	**.0006**	1.9 (1.1–3.0)	.01	2.0 (1.2–3.6)	.01
Lifetime illicit drug use ^f^	0.6 (0.5–0.8)	< .0001	0.7 (0.5–0.8)	**.0005**	1.4 (0.8–2.2)	.20	1.1 (0.7–2.0)	.65

All models adjusted for age, partnership status, employment status, K-12 harassment, and having experienced gender identity conversion efforts.

Abbreviations: OR (odds ratio), aOR (adjusted odds ratio), 95% CI (95% confidence interval).

^a^ Model also adjusted for gender identity, sex assigned at birth, sexual orientation, race/ethnicity, family support of gender identity, educational attainment, and total household income.

^b ^Model also adjusted for sexual orientation, race/ethnicity, educational attainment, and total household income.

^c^ Model also adjusted for gender identity, sex assigned at birth, sexual orientation, race/ethnicity, family support of gender identity, educational attainment, total household income, having received pubertal suppression.

^d^ Model also adjusted for family support of gender identity.

^e^ Model also adjusted for gender identity, sex assigned at birth, sexual orientation, family support of gender identity, educational attainment, and total household income.

^f ^Model also adjusted for gender identity, sex assigned at birth, sexual orientation, race/ethnicity, family support of gender identity, and educational attainment.

**Table 5:** Lifetime but no past-year suicide ideation and attempts for those who received gender-affirming hormones (estrogen or testosterone).

	Participants who Accessed GAH N = 12,598					
	Accessed GAH at Ages 13–15 n = 99		Accessed GAH at Ages 16 or 17 n = 362		Accessed GAH at Age ≥ 18 n = 12,257	
	aOR (95% CI)	p	aOR (95% CI)	p	aOR (95% CI)	p
Lifetime suicidal ideation and no past-year ideation ^a^	1.4 (0.9–2.2)	.17	1.4 (1.1–1.8)	.005	1.4 (1.3–1.5)	**< .0001**
Lifetime suicide attempt and no past-year attempt ^b^	0.9 (0.5–1.4)	.54	0.7 (0.6–1.0)	.03	1.0 (0.9–1.1)	.67

Mental health outcomes of transgender adults who recalled access to gender-affirming hormones (GAH) during various age groups. Reference group for all analyses is those who desired GAH but did not receive them. Both models adjusted for age, partnership status, employment status, K-12 harassment, and having experienced gender identity conversion efforts.

^a^ Model also adjusted for gender identity, sex assigned at birth, sexual orientation, race/ethnicity, family support of gender identity, educational attainment, and total household income.

^b^ Model also adjusted for gender identity, sex assigned at birth, sexual orientation, race/ethnicity, family support of gender identity, educational attainment, total household income, having received pubertal suppression.

These errors do not affect the results of the primary analyses or conclusions reported in the article.

Finally, the analysis code underlying results in this article was not included with the published article. With this Correction, the authors provide the code as S1 File.

A member of *PLOS ONE*’s Editorial Board confirmed that the new results support the results and conclusions of the published article.

The authors apologize for the errors in the published article.

## Supporting information

S1 Table Detailed in-text corrections. (DOCX)

S1 File Analysis code. (DOCX)
