# Supplementary material for: Correction: Access to gender-affirming hormones during adolescence and mental health outcomes among transgender adults
Source: PLoS One. 2023 Jun 12;18(6):e0287283. doi: 10.1371/journal.pone.0287283 (PMC10259783; doi:10.1371/journal.pone.0287283)
Supplement: S1 Table — (DOCX) [file pone.0287283.s001.docx]

| **Location of Error** | **Corrected Text** |
| --- | --- |
| Results section of the Abstract | 21,578 participants (77.9%) reported ever desiring GAH. Of these, 8,860 (41.0%) never accessed GAH, 99 (0.5%) accessed GAH in early adolescence, 362 (1.7%) accessed GAH in late adolescence, and 12,275 (56.8%) accessed GAH in adulthood. After adjusting for potential confounders, accessing GAH during early adolescence (aOR = 0.4, 95% CI = 0.3-0.6, p < .0001), late adolescence (aOR = 0.5, 95% CI = 0.4-0.7, p < .0001), or adulthood (aOR = 0.8, 95% CI = 0.7-0.8, p < .0001) was associated with lower odds of past-year suicidal ideation when compared to desiring but never accessing GAH. In post hoc analyses, access to GAH during adolescence (ages 13-17) was associated with lower odds of past-month severe psychological distress (aOR = 0.6, 95% CI = 0.5-0.8, p = .0002), compared to accessing GAH during adulthood. |
| Third paragraph of the Introduction, fifth and sixth sentence | More recent guidelines state that initiation of GAH can be considered as early as age 13.5, to allow transgender adolescents to undergo puberty at ages more comparable to their peers, and to reduce the risk of delayed bone development due to prolonged pubertal suppression [7]. In this article, we therefore consider two age groups of adolescents who initiated GAH: those who started GAH during late adolescence (i.e., between their 16th and 18th birthdays), and those who started GAH during early adolescence (i.e., between their 13th and 16th birthdays). |
| Third sentence of the ‘Age of initiation of GAH’ subsection in the Methods | The second group consisted of participants who reported they first received GAH during early adolescence, defined as the period between their 13th and 16th birthdays (GAH 13-16), which corresponds to the age group most recently added to the Endocrine Society Guidelines. |
| Last paragraph of the Methods section | In these post hoc analyses, we compared access to GAH during adolescence (ages 13-17) to access during adulthood (ages ≥18), and access during early adolescence (ages 13-15) to access during late adolescence (ages 16-17). |
| The ‘GAH during early adolescence’ subsection of the Results | The median age of participants who reported receiving GAH during early adolescence was 19.0 (IQR 18.0-30.0). After adjusting for demographic and potential confounding variables, recalled access to GAH during early adolescence was associated with lower odds of past-month severe psychological distress (aOR = 0.3, 95% CI = 0.2-0.5, p < .0001) and past-year suicidal ideation (aOR = 0.4, 95% CI = 0.3-0.6, p = .0001) when compared with those who desired GAH but never accessed them. These results indicate that the odds of past-month severe psychological distress and past-year suicide ideation for those who recalled access in early adolescence were decreased by 254% and 146%, respectively. We detected no difference for other mental health variables measured (Table 2). |
| The first sentence of the ‘GAH during adolescence vs. GAH during adulthood’ subsection of the results | After adjusting for demographic and potentially confounding variables, access to GAH during adolescence (ages 13-17 was associated with a lower odds of past-month severe psychological distress (aOR = 0.6, 95% CI = 0.5-0.8, p < .0002), past-year suicidal ideation (aOR = 0.7, 95% CI = 0.6-0.9, p = .004), past-month binge drinking (aOR = 0.6, 95% CI = 0.5-0.8, p = .0006), and lifetime illicit drug use (aOR = 0.7, 95% CI = 0.5-0.8, p = .0005) when compared to access to GAH during adulthood. |
