# Supplementary material for: Correction: Access to gender-affirming hormones during adolescence and mental health outcomes among transgender adults
Source: PLoS One. 2023 Jun 12;18(6):e0287283. doi: 10.1371/journal.pone.0287283 (PMC10259783; doi:10.1371/journal.pone.0287283)
Supplement: S1 File — (DOCX) [file pone.0287283.s002.docx]

/* Descriptives for Table 1 Sample demographics */

libname gahdir '\\fchc-datateam\Data\PROJECTS\Transgender Projects\NCTE\GAH\';

options fmtsearch=(gahdir library);

proc freq data=gahdir.GAH2 order=formatted;

tables Age4catCensus*AgeHTStart2 / fisher;

proc freq data=gahdir.GAH2 order=formatted;

tables gender5*AgeHTStart2 / fisher;

proc freq data=gahdir.GAH2 order=formatted;

tables BirthSex*AgeHTStart2 / chisq;

proc freq data=gahdir.GAH2 order=formatted;

tables q2_8cat7*AgeHTStart2 / chisq;

proc freq data=gahdir.GAH2 order=formatted;

tables raceacs2*AgeHTStart2 / chisq;

proc freq data=gahdir.GAH2 order=formatted;

tables famsupport3_recode*AgeHTStart2 / chisq;

proc freq data=gahdir.GAH2 order=formatted;

tables partnered_refyes*AgeHTStart2 / chisq;

proc freq data=gahdir.GAH2 order=formatted;

tables ed4_refBachelors*AgeHTStart2 / chisq;

proc freq data=gahdir.GAH2 order=formatted;

tables empstatus_recode*AgeHTStart2 / chisq;

proc freq data=gahdir.GAH2 order=formatted;

tables hhincome6*AgeHTStart2 / chisq;

proc freq data=gahdir.GAH2 order=formatted;

tables q12_9_3_recode*AgeHTStart2 / chisq;

proc freq data=gahdir.GAH2 order=formatted;

tables q13_2_recode*AgeHTStart2 / chisq;

proc freq data=gahdir.GAH2 order=formatted;

tables K12HARASSANY*AgeHTStart2 / chisq;

run;

/* Table 2 Outcomes by age groups for GAH start: Multivariable models */

libname gahdir '\\fchc-datateam\Data\PROJECTS\Transgender Projects\NCTE\GAH\';

options fmtsearch=(gahdir library);

/* psych distress model - all covariates */

proc logistic data = gahdir.GAH2;

class kessler6_YN (ref = "0") AgeHTStart2 (ref = "0. Wanted but never received GAH") / param = ref;

model kessler6_YN = AgeHTStart2 Age4catCensus gender5 BirthSex q2_8cat7 raceacs2 famsupport3_recode

partnered_refyes ed4_refBachelors empstatus_recode hhincome6_recode q12_9_3_recode q13_2_recode K12HARASSANY

/ link = glogit clodds=wald orpvalue;

/* past year SI - all covariates except PB blockers */

proc logistic data = gahdir.GAH2;

class PastYearSuicidality_YN (ref = "0") AgeHTStart2 (ref = "0. Wanted but never received GAH") / param = ref;

model PastYearSuicidality_YN = AgeHTStart2 BirthSex Age4catCensus gender5 q2_8cat7 raceacs2 famsupport3_recode

partnered_refyes ed4_refBachelors empstatus_recode hhincome6_recode q13_2_recode K12HARASSANY

/ link = glogit clodds=wald orpvalue;

/* SuicidePlanPastYear - gender, BirthSex, family support & PB blockers not sig with this outcome */

proc logistic data = gahdir.GAH2;

class SuicidePlanPastYear (ref = "0") AgeHTStart2 (ref = "0. Wanted but never received GAH") / param = ref;

model SuicidePlanPastYear = AgeHTStart2 Age4catCensus q2_8cat7 raceacs2

partnered_refyes ed4_refBachelors empstatus_recode hhincome6_recode q13_2_recode K12HARASSANY

/ link = glogit clodds=wald orpvalue;

/* Past Year Suicide Attempt - all covariates */

proc logistic data = gahdir.GAH2;

class PastYearSuicideAttempt_YN (ref = "0") AgeHTStart2 (ref = "0. Wanted but never received GAH") / param = ref;

model PastYearSuicideAttempt_YN = AgeHTStart2 Age4catCensus gender5 BirthSex q2_8cat7 raceacs2 famsupport3_recode

partnered_refyes ed4_refBachelors empstatus_recode hhincome6_recode q12_9_3_recode q13_2_recode K12HARASSANY

/ link = glogit clodds=wald orpvalue;

run;

/* HospStaySuicideAttemptRecode - limited covariates were sig */

proc logistic data = gahdir.GAH2;

class HospStaySuicideAttemptRecode (ref = "0") AgeHTStart2 (ref = "0. Wanted but never received GAH") / param = ref;

model HospStaySuicideAttemptRecode = AgeHTStart2 Age4catCensus famsupport3_recode partnered_refyes K12HARASSANY

empstatus_recode q13_2_recode / link = glogit clodds=wald orpvalue;

/* Past Month Binge Drinking - limited covariates were sig */

proc logistic data = gahdir.GAH2;

class PastMonthBingeDrinking (ref = "0") AgeHTStart2 (ref = "0. Wanted but never received GAH") / param = ref;

model PastMonthBingeDrinking = AgeHTStart2 Age4catCensus gender5 BirthSex q2_8cat7 famsupport3_recode

partnered_refyes ed4_refBachelors empstatus_recode hhincome6_recode K12HARASSANY / link = glogit clodds=wald orpvalue;

/* Lifetime Drug Use - limited covariates were sig */

proc logistic data = gahdir.GAH2;

class LifetimeDrugUse (ref = "0") AgeHTStart2 (ref = "0. Wanted but never received GAH") / param = ref;

model LifetimeDrugUse = AgeHTStart2 Age4catCensus gender5 BirthSex q2_8cat7 raceacs2 famsupport3_recode

partnered_refyes ed4_refBachelors empstatus_recode q13_2_recode K12HARASSANY / link = glogit clodds=wald orpvalue;

run;

/* Univariate analyses for mental health outcomes for 3 age groups who recived GAH compared to those who wanted GAH but never accessed them */

libname gahdir '\\fchc-datateam\Data\PROJECTS\Transgender Projects\NCTE\GAH\';

options fmtsearch=(gahdir library);

proc logistic data=gahdir.GAH2;

class kessler6_YN (ref = "0") AgeHTStart2 (ref = "0. Wanted but never received GAH") / param = ref;

model kessler6_YN = AgeHTStart2 / link = glogit clodds=wald orpvalue;

proc logistic data=gahdir.GAH2;

class PastYearSuicidality_YN (ref = "0") AgeHTStart2 (ref = "0. Wanted but never received GAH") / param = ref;

model PastYearSuicidality_YN = AgeHTStart2 / link = glogit clodds=wald orpvalue;

proc logistic data=gahdir.GAH2;

class SuicidePlanPastYear (ref = "0") AgeHTStart2 (ref = "0. Wanted but never received GAH") / param = ref;

model SuicidePlanPastYear = AgeHTStart2 / link = glogit clodds=wald orpvalue;

proc logistic data=gahdir.GAH2;

class PastYearSuicideAttempt_YN (ref = "0") AgeHTStart2 (ref = "0. Wanted but never received GAH") / param = ref;

model PastYearSuicideAttempt_YN = AgeHTStart2 / link = glogit clodds=wald orpvalue;

proc logistic data=gahdir.GAH2;

class HospStaySuicideAttemptRecode (ref = "0") AgeHTStart2 (ref = "0. Wanted but never received GAH") / param = ref;

model HospStaySuicideAttemptRecode = AgeHTStart2 / link = glogit clodds=wald orpvalue;

proc logistic data=gahdir.GAH2;

class PastMonthBingeDrinking (ref = "0") AgeHTStart2 (ref = "0. Wanted but never received GAH") / param = ref;

model PastMonthBingeDrinking = AgeHTStart2 / link = glogit clodds=wald orpvalue;

proc logistic data=gahdir.GAH2;

class LifetimeDrugUse (ref = "0") AgeHTStart2 (ref = "0. Wanted but never received GAH") / param = ref;

model LifetimeDrugUse = AgeHTStart2 / link = glogit clodds=wald orpvalue;

run;

/* Table 3 Outcome frequencies */

libname gahdir '\\fchc-datateam\Data\PROJECTS\Transgender Projects\NCTE\GAH\';

options fmtsearch=(gahdir library);

proc freq data = gahdir.GAH2;

tables kessler6_YN*AgeHTStart2 / missing;

tables PastYearSuicidality_YN*AgeHTStart2 / missing;

tables SuicidePlanPastYear*AgeHTStart2 / missing;

tables PastYearSuicideAttempt_YN*AgeHTStart2 / missing;

tables HospStaySuicideAttemptRecode*AgeHTStart2 / missing;

tables PastMonthBingeDrinking*AgeHTStart2 / missing;

tables LifetimeDrugUse*AgeHTStart2 / missing;

run;

/* Univariate analyses for mental health outcomes for all adolecents who recived GAH compared to adults */

libname gahdir '\\fchc-datateam\Data\PROJECTS\Transgender Projects\NCTE\GAH\';

options fmtsearch=(gahdir library);

proc logistic data=gahdir.GAH2;

class kessler6_YN (ref = "0") AgeGrps_HTStart_TeensVSAdults (ref = FIRST) / param = ref;

model kessler6_YN = AgeGrps_HTStart_TeensVSAdults / link = glogit clodds=wald;

proc logistic data=gahdir.GAH2;

class PastYearSuicidality_YN (ref = "0") AgeGrps_HTStart_TeensVSAdults (ref = FIRST) / param = ref;

model PastYearSuicidality_YN = AgeGrps_HTStart_TeensVSAdults / link = glogit clodds=wald;

proc logistic data=gahdir.GAH2;

class SuicidePlanPastYear (ref = "0") AgeGrps_HTStart_TeensVSAdults (ref = FIRST) / param = ref;

model SuicidePlanPastYear = AgeGrps_HTStart_TeensVSAdults / link = glogit clodds=wald;

proc logistic data=gahdir.GAH2;

class PastYearSuicideAttempt_YN (ref = "0") AgeGrps_HTStart_TeensVSAdults (ref = FIRST) / param = ref;

model PastYearSuicideAttempt_YN = AgeGrps_HTStart_TeensVSAdults / link = glogit clodds=wald;

proc logistic data=gahdir.GAH2;

class HospStaySuicideAttemptRecode (ref = "0") AgeGrps_HTStart_TeensVSAdults (ref = FIRST) / param = ref;

model HospStaySuicideAttemptRecode = AgeGrps_HTStart_TeensVSAdults / link = glogit clodds=wald;

proc logistic data=gahdir.GAH2;

class PastMonthBingeDrinking (ref = "0") AgeGrps_HTStart_TeensVSAdults (ref = FIRST) / param = ref;

model PastMonthBingeDrinking = AgeGrps_HTStart_TeensVSAdults / link = glogit clodds=wald;

proc logistic data=gahdir.GAH2;

class LifetimeDrugUse (ref = "0") AgeGrps_HTStart_TeensVSAdults (ref = FIRST) / param = ref;

model LifetimeDrugUse = AgeGrps_HTStart_TeensVSAdults / link = glogit clodds=wald;

run;

/* Univariate models for mental health outcomes for group who started GAH as younger teens compared to older teens */

libname gahdir '\\fchc-datateam\Data\PROJECTS\Transgender Projects\NCTE\GAH\';

options fmtsearch=(gahdir library);

proc logistic data=gahdir.GAH2;

class kessler6_YN (ref = "0") AgeGrps_HTStart2Teens (ref = "0. GAH 16 or 17") / param = ref;

model kessler6_YN = AgeGrps_HTStart2Teens / link = glogit clodds=wald orpvalue;

proc logistic data=gahdir.GAH2;

class PastYearSuicidality_YN (ref = "0") AgeGrps_HTStart2Teens (ref = "0. GAH 16 or 17") / param = ref;

model PastYearSuicidality_YN = AgeGrps_HTStart2Teens / link = glogit clodds=wald orpvalue;

proc logistic data=gahdir.GAH2;

class SuicidePlanPastYear (ref = "0") AgeGrps_HTStart2Teens (ref = "0. GAH 16 or 17") / param = ref;

model SuicidePlanPastYear = AgeGrps_HTStart2Teens / link = glogit clodds=wald orpvalue;

proc logistic data=gahdir.GAH2;

class PastYearSuicideAttempt_YN (ref = "0") AgeGrps_HTStart2Teens (ref = "0. GAH 16 or 17") / param = ref;

model PastYearSuicideAttempt_YN = AgeGrps_HTStart2Teens / link = glogit clodds=wald orpvalue;

proc logistic data=gahdir.GAH2;

class HospStaySuicideAttemptRecode (ref = "0") AgeGrps_HTStart2Teens (ref = "0. GAH 16 or 17") / param = ref;

model HospStaySuicideAttemptRecode = AgeGrps_HTStart2Teens / link = glogit clodds=wald orpvalue;

proc logistic data=gahdir.GAH2;

class PastMonthBingeDrinking (ref = "0") AgeGrps_HTStart2Teens (ref = "0. GAH 16 or 17") / param = ref;

model PastMonthBingeDrinking = AgeGrps_HTStart2Teens / link = glogit clodds=wald orpvalue;

proc logistic data=gahdir.GAH2;

class LifetimeDrugUse (ref = "0") AgeGrps_HTStart2Teens (ref = "0. GAH 16 or 17") / param = ref;

model LifetimeDrugUse = AgeGrps_HTStart2Teens / link = glogit clodds=wald orpvalue;

run;

/* Multivariable models for mental health outcomes for group who started GAH as teens compared to adults */

libname gahdir '\\fchc-datateam\Data\PROJECTS\Transgender Projects\NCTE\GAH\';

options fmtsearch=(gahdir library);

/* psych distress model - all covariates */

proc logistic data = gahdir.GAH2;

class kessler6_YN (ref = "0") AgeGrps_HTStart_TeensVSAdults (ref = FIRST) / param = ref;

model kessler6_YN = AgeGrps_HTStart_TeensVSAdults Age4catCensus gender5 BirthSex q2_8cat7 raceacs2 famsupport3_recode

partnered_refyes ed4_refBachelors empstatus_recode hhincome6_recode q12_9_3_recode q13_2_recode K12HARASSANY

/ link = glogit clodds=wald orpvalue;

/* past year SI - all covariates except PB blockers */

proc logistic data = gahdir.GAH2;

class PastYearSuicidality_YN (ref = "0") AgeGrps_HTStart_TeensVSAdults (ref = FIRST) / param = ref;

model PastYearSuicidality_YN = AgeGrps_HTStart_TeensVSAdults Age4catCensus gender5 BirthSex q2_8cat7 raceacs2 famsupport3_recode

partnered_refyes ed4_refBachelors empstatus_recode hhincome6_recode q13_2_recode K12HARASSANY

/ link = glogit clodds=wald orpvalue;

/* SuicidePlanPastYear - gender, BirthSex, family support & PB blockers not sig with this outcome */

proc logistic data = gahdir.GAH2;

class SuicidePlanPastYear (ref = "0") AgeGrps_HTStart_TeensVSAdults (ref = FIRST) / param = ref;

model SuicidePlanPastYear = AgeGrps_HTStart_TeensVSAdults Age4catCensus q2_8cat7 raceacs2

partnered_refyes ed4_refBachelors empstatus_recode hhincome6_recode q13_2_recode K12HARASSANY

/ link = glogit clodds=wald orpvalue;

/* Past Year Suicide Attempt - all covariates */

proc logistic data = gahdir.GAH2;

class PastYearSuicideAttempt_YN (ref = "0") AgeGrps_HTStart_TeensVSAdults (ref = FIRST) / param = ref;

model PastYearSuicideAttempt_YN = AgeGrps_HTStart_TeensVSAdults Age4catCensus gender5 BirthSex q2_8cat7 raceacs2 famsupport3_recode

partnered_refyes ed4_refBachelors empstatus_recode hhincome6_recode q12_9_3_recode q13_2_recode K12HARASSANY

/ link = glogit clodds=wald orpvalue;

/* HospStaySuicideAttemptRecode - limited covariates were sig */

proc logistic data = gahdir.GAH2;

class HospStaySuicideAttemptRecode (ref = "0") AgeGrps_HTStart_TeensVSAdults (ref = FIRST) / param = ref;

model HospStaySuicideAttemptRecode = AgeGrps_HTStart_TeensVSAdults Age4catCensus famsupport3_recode partnered_refyes

empstatus_recode q13_2_recode K12HARASSANY / link = glogit clodds=wald orpvalue;

/* Past Month Binge Drinking - limited covariates were sig */

proc logistic data = gahdir.GAH2;

class PastMonthBingeDrinking (ref = "0") AgeGrps_HTStart_TeensVSAdults (ref = FIRST) / param = ref;

model PastMonthBingeDrinking = AgeGrps_HTStart_TeensVSAdults Age4catCensus gender5 BirthSex q2_8cat7 famsupport3_recode

partnered_refyes ed4_refBachelors empstatus_recode hhincome6_recode K12HARASSANY / link = glogit clodds=wald orpvalue;

/* Lifetime Drug Use - limited covariates were sig */

proc logistic data = gahdir.GAH2;

class LifetimeDrugUse (ref = "0") AgeGrps_HTStart_TeensVSAdults (ref = FIRST) / param = ref;

model LifetimeDrugUse = AgeGrps_HTStart_TeensVSAdults Age4catCensus gender5 BirthSex q2_8cat7 raceacs2 famsupport3_recode

partnered_refyes ed4_refBachelors empstatus_recode q13_2_recode K12HARASSANY / link = glogit clodds=wald orpvalue;

run;

/* Mutivariable models for mental health outcomes comparing the groups who started GAH as younger vs. older teens */

libname gahdir '\\fchc-datateam\Data\PROJECTS\Transgender Projects\NCTE\GAH\';

options fmtsearch=(gahdir library);

/* psych distress model - all covariates */

proc logistic data = gahdir.GAH2;

class kessler6_YN (ref = "0") AgeGrps_HTStart2Teens (ref = "0. GAH 16 or 17") / param = ref;

model kessler6_YN = AgeGrps_HTStart2Teens Age4catCensus gender5 BirthSex q2_8cat7 raceacs2 famsupport3_recode

partnered_refyes ed4_refBachelors empstatus_recode hhincome6_recode q12_9_3_recode q13_2_recode K12HARASSANY

/ link = glogit clodds=wald orpvalue;

run;

/* past year SI - all covariates except PB blockers */

proc logistic data = gahdir.GAH2;

class PastYearSuicidality_YN (ref = "0") AgeGrps_HTStart2Teens (ref = "0. GAH 16 or 17") / param = ref;

model PastYearSuicidality_YN = AgeGrps_HTStart2Teens Age4catCensus gender5 BirthSex q2_8cat7 raceacs2 famsupport3_recode

partnered_refyes ed4_refBachelors empstatus_recode hhincome6_recode q13_2_recode K12HARASSANY

/ link = glogit clodds=wald orpvalue;

run;

/* SuicidePlanPastYear - gender, BirthSex, family support & PB blockers not sig with this outcome */

proc logistic data = gahdir.GAH2;

class SuicidePlanPastYear (ref = "0") AgeGrps_HTStart2Teens (ref = "0. GAH 16 or 17") / param = ref;

model SuicidePlanPastYear = AgeGrps_HTStart2Teens Age4catCensus q2_8cat7 raceacs2

partnered_refyes ed4_refBachelors empstatus_recode hhincome6_recode q13_2_recode K12HARASSANY

/ link = glogit clodds=wald orpvalue;

run;

/* Past Year Suicide Attempt - all covariates */

proc logistic data = gahdir.GAH2;

class PastYearSuicideAttempt_YN (ref = "0") AgeGrps_HTStart2Teens (ref = "0. GAH 16 or 17") / param = ref;

model PastYearSuicideAttempt_YN = AgeGrps_HTStart2Teens Age4catCensus gender5 BirthSex q2_8cat7 raceacs2 famsupport3_recode

partnered_refyes ed4_refBachelors empstatus_recode hhincome6_recode q12_9_3_recode q13_2_recode K12HARASSANY

/ link = glogit clodds=wald orpvalue;

run;

/* HospStaySuicideAttemptRecode - limited covariates were sig */

proc logistic data = gahdir.GAH2;

class HospStaySuicideAttemptRecode (ref = "0") AgeGrps_HTStart2Teens (ref = "0. GAH 16 or 17") / param = ref;

model HospStaySuicideAttemptRecode = AgeGrps_HTStart2Teens Age4catCensus famsupport3_recode partnered_refyes

empstatus_recode q13_2_recode K12HARASSANY / link = glogit clodds=wald orpvalue;

run;

/* Past Month Binge Drinking - limited covariates were sig */

proc logistic data = gahdir.GAH2;

class PastMonthBingeDrinking (ref = "0") AgeGrps_HTStart2Teens (ref = "0. GAH 16 or 17") / param = ref;

model PastMonthBingeDrinking = AgeGrps_HTStart2Teens Age4catCensus gender5 BirthSex q2_8cat7 famsupport3_recode

partnered_refyes ed4_refBachelors empstatus_recode hhincome6_recode K12HARASSANY / link = glogit clodds=wald orpvalue;

run;

/* Lifetime Drug Use - limited covariates were sig */

proc logistic data = gahdir.GAH2;

class LifetimeDrugUse (ref = "0") AgeGrps_HTStart2Teens (ref = "0. GAH 16 or 17") / param = ref;

model LifetimeDrugUse = AgeGrps_HTStart2Teens Age4catCensus gender5 BirthSex q2_8cat7 raceacs2 famsupport3_recode

partnered_refyes ed4_refBachelors empstatus_recode q13_2_recode K12HARASSANY / link = glogit clodds=wald orpvalue;

run;

/* Table 5: Post hoc tests for SI Lifetime but no past year, including all covariates except puberty blockers */

libname gahdir '\\fchc-datateam\Data\PROJECTS\Transgender Projects\NCTE\GAH\';

options fmtsearch=(gahdir library);

proc logistic data = gahdir.GAH2;

class SI_LifetimeNoPastYear (ref = "0") AgeHTStart2 (ref = "0. Wanted but never received GAH") / param = ref;

model SI_LifetimeNoPastYear = AgeHTStart2 Age4catCensus gender5 BirthSex q2_8cat7 raceacs2 famsupport3_recode

partnered_refyes ed4_refBachelors empstatus_recode hhincome6_recode q13_2_recode K12HARASSANY

/ link = glogit clodds=wald orpvalue;

/* Suicide Attempt Lifetime but no past year - all covariates except partner status & puberty blockers */

proc logistic data = gahdir.GAH2;

class Attempt_LifetimeNoPastYear (ref = "0") AgeHTStart2 (ref = "0. Wanted but never received GAH") / param = ref;

model Attempt_LifetimeNoPastYear = AgeHTStart2 Age4catCensus gender5 BirthSex q2_8cat7 raceacs2 famsupport3_recode

ed4_refBachelors empstatus_recode hhincome6_recode q13_2_recode K12HARASSANY

/ link = glogit clodds=wald orpvalue;

run;
